# Global Evolution of Robotic Colorectal Surgery: Lessons from Hong Kong’s Innovation and Implementation

**DOI:** 10.3390/cancers18081259

**Published:** 2026-04-16

**Authors:** Trevor M. Yeung, Justin N. F. Lam, Rossetti H. Y. Lam, Simon S. Ng

**Affiliations:** Division of Colorectal Surgery, Department of Surgery, The Chinese University of Hong Kong, Prince of Wales Hospital, Shatin, Hong Kong SAR, China

**Keywords:** robotic colorectal surgery, innovation, training, governance

## Abstract

Robotic colorectal surgery has revolutionized minimally invasive techniques worldwide, offering a more stable operative platform, superior 3D visualization, and wristed instrumentation for challenging pelvic dissections. This narrative review describes the global evolution of robotic colorectal surgery, from the use of multi-port to single-port systems, modular platforms and endoluminal robotic devices. Using Hong Kong’s role as an innovation and research hub, this review demonstrates how integrated innovation, structured training, workflow efficiencies and digital policy frameworks can overcome barriers and inform international implementation of robotic colorectal surgery. Since 2005, The Chinese University of Hong Kong has been pioneering the use of robotic platforms in colorectal surgery, performing first-in-human trials of the da Vinci SP system, the locally developed Sentire C1000 and the EndoMaster EASE for robotic ESD. There is increasing evidence supporting the use of the robotic platform over laparoscopic colorectal surgery, with benefits including reduced open conversions, fewer intraoperative complications, shorter hospital stay, better long-term functional outcomes and improved oncologic outcomes. However, several challenges remain before robotics can be implemented widely on a global scale, such as higher initial capital costs, limited training access, surgeon credentialing and governance for AI-driven data analytics.

## Simple Summary

The development of multiple robotic platforms has transformed how surgeons perform minimally invasive colorectal procedures by providing a stable view, precise tools with flexible wrists, and better access to tight spaces in the pelvis. This review highlights the progress and development of these robotic systems, from multi-arm setups to single-port and flexible designs. Using Hong Kong’s role as an international research hub, we describe how innovation, structured learning and data governance can overcome hurdles in the wider implementation of robotic surgery and lead to tangible benefits to patients, surgeons and healthcare systems globally.

## 1. Introduction

Over the past two decades, robotic colorectal surgery has expanded rapidly worldwide, transforming the delivery of minimally invasive surgery and highlighting clear technical advantages of robotic platforms. Whilst the development of laparoscopic surgery undoubtedly allows for magnified views of the rectum and pelvis [1] compared with open surgery, robotic surgery provides three-dimensional views on a stable platform and greater comfort to the surgeon during long challenging procedures [2]. In Hong Kong, early adoption and sustained innovation have positioned CUHK and the Prince of Wales Hospital (PWH) as leaders in the field of robotic colorectal surgery. Since installation of the first da Vinci system at PWH in 2005, our unit has progressed from initial feasibility work to complex pelvic procedures and multi-platform utilisation. Unlike prior platform-focused reviews, this article uniquely reviews the global evolution of robotic colorectal surgery using Hong Kong as a case study to illustrate how innovation, training and governance can be integrated within a public healthcare system, and contrasts this with the focus on high-volume private sector penetration in the US, the use of modular platforms in Europe, and the preference for domestic platforms in Japan.

## 2. Innovation of Robotic Colorectal Surgery in Hong Kong

**Figure 1:**
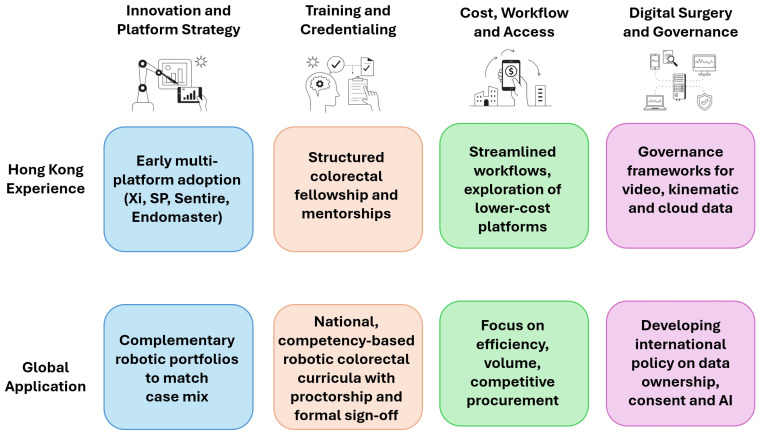
Lessons from Hong Kong’s robotic colorectal surgery programme and their application to global implementation. This figure summarises how Hong Kong’s experience in innovation and platform strategy, training and credentialing, cost and workflow, and digital surgery and governance can inform international implementation of robotic colorectal surgery.

Internationally, the development of robotic colorectal surgery has progressed from early multi-port systems to newer single-port, modular multi-platform and flexible endoluminal robots, reflecting a shared drive to enhance precision while minimising access trauma. Within this wider evolution, Hong Kong has emerged as an international innovation hub. Over the past two decades, CUHK/PWH has contributed to several first-in-human and early-phase clinical trials in robotic colorectal surgery, including novel approaches to single-port surgery and robotic endoscopic submucosal dissection (ESD). These programmes, often conducted in collaboration with industry partners and international centres, have allowed Hong Kong to act as a technological and innovation hub for next-generation robotic platforms and workflows (Figure 1).

The da Vinci Single-Port (SP) platform represents the next evolution of robotic colorectal surgery, building on the advances of multi-port systems while aiming to minimise access-related trauma. Unlike earlier platforms with four independent arms, the SP system delivers a flexible 3D endoscope and three multi-jointed, wristed instruments through a single 25 mm cannula, enabling true single-incision transabdominal surgery and facilitating transanal access to the narrow pelvis. CUHK conducted the world’s first multispecialty SP clinical trial including colorectal and transoral robotic surgery, demonstrating feasibility and safety in selected patients [3]. Further studies on SP colorectal surgery have reported low conversion rates, acceptable morbidity, negative resection margins and adequate lymph node yields [4,5,6,7,8].

SP colorectal surgery offers several potential advantages: simplified docking, improved cosmetic outcomes, and dual-articulating instruments that allow effective intracorporeal triangulation even in a narrow pelvis, which is particularly attractive for low rectal and transanal procedures. However, limitations such as a restricted portfolio of compatible instruments, reach constraints for high splenic flexure mobilisation and the need for careful case selection mean that SP is currently best suited for pelvic procedures or robotic transanal minimally invasive surgery (TAMIS).

In parallel with international work on single-port robotics, CUHK has also contributed to the development and clinical evaluation of the locally developed Cornerstone Sentire Robotic Surgical System (C1000), a multi-arm robotic platform designed for multispecialty minimally invasive surgery. Functionally, the Sentire system has been designed to mirror many of the core capabilities of established multi-arm robotic platforms such as the da Vinci Xi, offering a four-arm cart, three-dimensional high-definition vision and wristed instruments controlled from an immersive surgeon console, with a broadly familiar console layout and instrument kinematics that allow surgeons with prior Xi experience to transition with minimal retraining. By targeting a substantially lower overall cost of acquisition and use, Sentire explicitly aims to deliver a more accessible model of robotic surgery within publicly funded healthcare systems, while early pilot and prospective studies suggest that it can achieve clinical outcomes comparable to existing robotic platforms in major gastrointestinal and urologic procedures [9]. The development of Sentire mirrors a broader global trend towards multi-vendor robotic ecosystems, with newer platforms such as Versius [10,11], Hugo [12,13] and Hinotori [14,15] seeking to introduce cost competition and platform diversity (Table 1).

Building on CUHK’s experience with the development of flexible endoluminal platforms, a prospective phase II single-arm clinical trial at Prince of Wales Hospital evaluated colonic ESD using the EndoMaster EASE system, a flexible robotic platform with two independently controlled arms designed to improve tissue traction and dissection stability. In 43 patients with early colorectal neoplasia, robotic ESD achieved technical success in 86%, with an en bloc resection rate of 94.6% among technically successful cases, a complete (R0) resection rate of 83.8%, and a median specimen size of 35 mm. Procedure efficiency and safety were favourable, with a median robotic dissection time of 49 min, median hospital stay of 2 days, one delayed bleed and one perforation both managed endoscopically without sequelae. With a median 24-month follow-up, no local recurrences were detected, supporting the feasibility and safety of flexible robotic ESD for appropriately selected colorectal lesions [16]. The Hong Kong experience with EndoMaster illustrates an emerging international paradigm in which organ-preserving endoluminal robotics are beginning to complement abdominal platforms rather than replacing them.

## 3. Robotic Colorectal Surgery Benefits Patients

Robotic surgery may offer tangible benefits beyond laparoscopy particularly for technically demanding rectal procedures, though evidence remains heterogeneous especially for colonic resections and pilot data for newer platforms, such as SP and Sentire, lack long-term oncologic outcomes. Robotic platforms enhance precision in the deep pelvis, allowing stable three-dimensional vision and wristed instruments to deliver more accurate dissection, better preservation of mesorectal planes and more reliable margins, while maintaining the advantages of minimally invasive surgery such as smaller incisions, less pain and reduced blood loss. In the REAL multi-centre randomised controlled trial comparing robotic total mesorectal excision (RTME) with laparoscopic TME (LTME), RTME was associated with better postoperative gastrointestinal recovery, shorter postoperative hospital stay, fewer abdominoperineal resections, fewer conversions to open surgery, reduced blood loss and fewer intraoperative complications [17]. Long-term outcomes included lower 3-year locoregional recurrence, higher 3-year disease-free survival rates, and improved urinary, sexual and defecation function after surgery in the robotic group [18].

A separate single-centre randomised trial comparing laparoscopic and robotic abdominoperineal resection (LAPR vs. RAPR) similarly demonstrated reduced postoperative complication rate, reduced open conversion rate, less intraoperative bleeding, reduced 30-day readmission rate and reduced hospital stay in patients with low rectal cancer, underscoring the value of robotics in technically demanding pelvic surgery [19]. The COLRAR trial, which was terminated early because of poor accrual related to patient preference for robotics, did not show an overall superiority of RTME over LTME, but subgroup analyses showed that positive CRM rates were lower in the RTME group consistent with improved surgical precision [20].

Functional outcomes also appear to favour robotics: a meta-analysis by Yang et al. reported better short-term International Prostate Symptom Scores and sexual function indices after RTME compared with LTME [21], while Fleming et al. found improved urinary and erectile function scores in men undergoing robotic versus laparoscopic rectal cancer surgery [22]. Taken together, these data indicate that for appropriately selected patients with mid and low rectal cancer, robotic surgery can enhance oncological quality, reduce complications and support better preservation of urinary and sexual function compared with conventional laparoscopy. However, these data should be interpreted in the context of potential centre/patient selection bias, learning curve effects, and cost–benefit ratios that remain debated.

Evidence for robotic colorectal surgery is most robust in rectal cancer, whereas data for colonic disease remain less mature and are largely derived from retrospective series and prospective cohorts. Nonetheless, robotic platforms appear particularly promising for technically demanding colonic procedures such as complete mesocolic excision, where enhanced three-dimensional vision and stable articulation may support precise central vascular ligation and high-quality mesocolic planes. For robotic colonic resections, the stable platform also facilitates intracorporeal anastomosis with the potential for reduced mesenteric traction and smaller extraction sites, which could lead to a reduction in postoperative ileus [23]. The use of indocyanine green (ICG) together with fluorescence imaging incorporated within the robotic platforms also offers the possibility of improved visualisation of lymph node and critical vessel dissection during colorectal surgery [24].

## 4. Robotic Surgery Benefits Surgeons Through Improved Ergonomics

The development of robotic platforms has helped to overcome some of the ergonomic challenges that are encountered during laparoscopic surgery. Due to the greater visualisation and the ease of manipulating surgical tissues and sutures through the articulated instruments, robotic platforms can help reduce incidences of muscular strain. Carpal tunnel syndrome and thenar neuropathy, both associated with prolonged use of laparoscopic pistol-grip instruments, have not been reported with the use of robotic surgery [25].

Wearable-sensor studies comparing conventional laparoscopy with robotic platforms have demonstrated that robot-assisted procedures allow surgeons to work in more ergonomic postures, with reduced upper-limb muscle loading and less shoulder abduction than during standard laparoscopy. Although trunk flexion was slightly greater at the console, this increase was typically under 15 degrees and was not considered highly detrimental when the console height and armrests were adjusted correctly. Overall improved surgeon posture was obtained with robotic surgery. supporting the view that robotic colorectal surgery can reduce cumulative musculoskeletal strain [26]. The alleviation of physical demands in robotic surgery can also enhance the quality of surgical care that is delivered as well. With the relief of pain and discomfort post-procedure, surgeries can be performed with greater attention and accuracy. By preserving surgeon wellbeing over their life-time career, this will help reduce overall healthcare costs associated with absenteeism due to occupational injuries, and reduce the need to train more replacement surgeons due to early retirement [23].

Ergonomic performance is not uniform across platforms. Closed-console systems with limited adjustability have historically posed challenges for very tall or short surgeons, who often had to adopt awkward neck and back positions to align with the eyepieces, whereas newer open-console designs used by systems such as Versius and Hugo offer greater flexibility in monitor and hand-control positioning, which has been associated with lower ergonomic risk scores [27] whilst maintaining team communication. More recent iterations of robotic platforms, including the da Vinci 5 (dV5) system [28], have been designed specifically to address many of the ergonomic and workflow limitations observed with earlier generations of the da Vinci system. Enhanced vision, instrument haptic feedback, improved console adjustability and better integration of auxiliary displays aim to reduce musculoskeletal strain for surgeons, streamline team communication and create a more conducive environment for teaching and training. The rapid introduction of platforms such as da Vinci 5, Versius, Hugo and other next-generation systems underscores a global recognition that ergonomics and workflow are central design priorities rather than secondary considerations

## 5. Training in Robotic Colorectal Surgery

Structured training programmes are essential to ensure that new robotic technologies are implemented safely and consistently for patients. In Hong Kong, generic robotic surgery training pathways are available through industry partners, typically combining online modules, simulator-based skills acquisition, dry and wet lab training, and proctored clinical cases on the platform. At CUHK/PWH, this is complemented by a dedicated colorectal robotic surgery training programme that integrates case selection, stepwise autonomy on the console and regular assessment of technical performance within a mentorship model. Despite these initiatives, there remains no territory-wide standardised colorectal robotic training and credentialing framework across all Hospital Authority hospitals and as yet, no colorectal robotic training programme in Hong Kong has been formally accredited by the College of Surgeons of Hong Kong. Currently, there are few opportunities for residents or trainees to access robotic surgical training on the console. Developing a coordinated, multi-centre curriculum with agreed benchmarks for competency and progression is therefore critical to ensure equitable access to robotic expertise and maintaining consistent oncological and functional outcomes [29].

Training with robotic systems involves two aspects: technical training and developing competence. In order to achieve both, currently established training programmes incorporate a multi-modal and component-based approach. These consist of theoretical knowledge, case observation, simulation and proctored training [30]. The theoretical knowledge block, often delivered in forms of lecture, webinar or online format allows for efficient mass entry into the process. Case observation is often undertaken prior to hands on console usage. To be more efficient, programmes have opted to observe a portion of cases from cross specialties such as urology instead of purely colorectal surgery cases [31]. All training programmes incorporate simulation into one of its steps. A combination of simulation, dry and wet lab training is employed to perform non-anatomical and simple surgical tasks [30]. Lastly, all programmes also involve proctored cases. Competency assessments are usually performed during the simulation and proctored cases with the most common standardised scoring system being European Academy of Robotic Colorectal Surgery (EARCS) Global Assessment Score (GAS) [30]. Limited access to robotic platforms is a common theme in different countries when trying to establish standardised training programmes. The Association of Coloproctology of Great Britain and Ireland encourages early exposure of robotic surgery to trainees with access to theoretical learning and also hands on with robotic simulators to build up their core robotic skills [32].

Currently, with limited training opportunities, only established surgeons and fellows have access to training on robotic platforms. This is logical since the resources needed to train the surgeons with a significant background in laparoscopic surgery is less. Prior laparoscopic training provides a significant advantage in theoretical and practical aspects, as these surgeons possess a better understanding of anatomical relationships and are more aware of instruments outside the direct field of view [31]. Simultaneous training delivered to multiple fellows concurrently is also possible within a structured, mentored training programme, whilst maintaining a high standard of clinical and oncological care [33].

## 6. Wider Implications in the Adoption of Robotic Colorectal Surgery

The drivers and challenges of adopting robotic colorectal surgery are remarkably similar across health systems, centering on cost-effectiveness, equitable access and the governance of increasingly data-rich digital platforms. The increasing adoption of robotic technology in colorectal surgery has generated discussion regarding its cost/benefit implications, ethical and regulatory challenges when compared to conventional laparoscopy. In a recent cohort study comparing robot-assisted to laparoscopic colorectal surgery, the median total cost of the surgery was US$16,628 in the robotic group compared with $14,641 in the laparoscopic group. Furthermore, the cost per day of robotic-assisted surgery was also more expensive at $3517 compared to $2961 for laparoscopic surgery [34]. Other studies have reported that the cost of robotic rectal surgery was estimated to be up to three times more expensive than laparoscopic rectal surgery [35]. Conversely, in a retrospective, single institution study, robotic-assisted low anterior resections were found to have significantly reduced cost (US$14,093 vs. $17,314), reduced operative time (311 min vs. 366 min), and reduced readmission rate (5.7% vs. 20.6%) compared to the laparoscopic group. Although this was performed in a single centre, the significance of this study lies in its implementation of a standardised surgical protocol with a dedicated OR team and streamlined workflow, resulting in reduced costs in robotic surgery [36]. Furthermore, the ROBOCOSTES study demonstrated that although robotic rectal surgery was associated with greater short term costs, it was superior in quality-adjusted life years compared to laparoscopic rectal surgery [37].

While current analyses often show higher per-case costs for robotic colorectal surgery compared with laparoscopy, particularly when capital expenditure and consumables are included, this picture is likely to evolve as more affordable platforms are commercialised and competition increases. A decision-analytic modelling study has suggested that robotic surgery can become more cost-effective than laparoscopic surgery if operative times are reduced and complication rates are sufficiently lower, underscoring the importance of optimising team efficiency and case selection rather than focusing on acquisition cost alone [38]. For Hong Kong, investment in training, streamlined theatre workflows and appropriate indication selection is as important as hardware pricing in determining the value proposition of robotics, and that periodic re-evaluation of cost-effectiveness will be necessary as platforms, volumes and outcomes continue to change.

Within the Hospital Authority, credentialing systems for robotic surgery and monitoring of operative outcomes are already in place, providing a basic framework to ensure that new robotic surgeons achieve defined case numbers and acceptable short-term results before independent practice. These mechanisms offer an important layer of governance and quality assurance as robotic indications expand across colorectal units in the territory. However, existing policies were largely developed for earlier generations of robotic surgery and do not yet fully address emerging issues such as the use of video and kinematic data for performance assessment and the possibility of remote telesurgery in future. Ethical risks of algorithmic bias from Western-dominated training data, data ownership disputes between surgeons and manufacturers, and real-world implementation barriers such as cross-platform data sharing further complicate governance.

Modern robotic platforms now capture rich streams of digital information during each procedure, including high-definition video, instrument kinematics, and system events. These datasets are frequently uploaded to centralised cloud or manufacturer-managed hubs, where they are aggregated and analysed using advanced analytics and artificial intelligence techniques to derive performance metrics, identify workflow bottlenecks and develop decision-support tools for future iterations of the technology. This shift towards “digital surgery” raises fundamental questions about data ownership, consent, cross-border data transfer and how surgeon-specific performance information should be used within credentialing and appraisal systems in Hong Kong. Clear governance frameworks will be required to balance the benefits of data-driven innovation against the need to protect patient privacy, maintain professional trust and align with local regulatory expectations. Comparable debates are emerging worldwide as national ‘digital surgery’ initiatives begin to incorporate automated performance metrics and AI-supported decision tools based on video and kinematic data. Hong Kong’s high digital maturity and concentration of robotic expertise mean that it could serve as a test bed for governance models that balance innovation with robust data protection and professional trust.

## 7. Conclusions and Future Directions

Robotic colorectal surgery in Hong Kong has progressed from early adoption to a mature service with active contributions in R&D, first-in-human trials and digital innovation, offering practical lessons for the global evolution of the field. Although current costs remain higher than laparoscopy, emerging platforms, optimised workflows and lower complication rates provide a realistic path towards cost-effective implementation. The key challenges now lie in establishing a territory-wide, college-endorsed training and credentialing framework and updating governance for data-rich, AI-enabled robotic platforms. By aligning investment in training, technology and policy, Hong Kong can act as an international model for safe, sustainable and value-driven implementation of robotic colorectal surgery.

## Figures and Tables

**Table 1:** Major and emerging robotic platforms used for colorectal surgery.

Platform	Console and Ergonomics	Arm/Port Configuration	Key Colorectal Applications	Notes on Cost and Access
**da Vinci Xi (Intuitive Surgical)**	Closed immersive console with 3D HD vision; adjustable seat and controls but closed design may still pose neck/back strain in very tall or short surgeons.	Multi-port system with four robotic arms on an overhead boom; 8 mm wristed instruments and integrated energy platforms.	Global workhorse for robotic TME, low anterior resection, extended colectomy and multi-quadrant procedures.	High capital and maintenance costs; dominant platform in North America, Europe and much of Asia, including Hong Kong public hospitals.
**da Vinci SP (Intuitive Surgical)**	Closed console similar to Xi with ergonomic hand controls; highly flexible 3D scope improves visualisation in deep, narrow pelvis and for transanal access.	Single 25 mm cannula housing a flexible 3D endoscope plus three multi-jointed wristed instruments, enabling single-incision and natural-orifice approaches.	Single-port colectomy, low rectal TME and hybrid transanal/SP-TME, particularly advantageous in narrow male pelvis.	Limited availability and indications; early adoption via structured trials in high-volume centres in Asia and the US, including CUHK/PWH.
**da Vinci 5 (Intuitive Surgical)**	Next-generation closed console with enhanced vision, improved haptic feedback and greater adjustability of armrests and display to reduce musculoskeletal strain and facilitate teaching.	Multi-port configuration broadly similar to Xi, with refinements in instrument technology and system responsiveness.	Designed for complex pelvic procedures and multi-quadrant colorectal surgery, with focus on precision and ergonomic improvements.	Early clinical experience suggests potential for reduced tissue trauma and more efficient operating room workflows; roll-out initially limited to selected high-volume centres.
**Versius (CMR Surgical)**	Open console, joystick-style controls and 3D monitor improve team communication and lower ergonomic risk scores.	Modular bedside arms on individual carts, enabling flexible port placement and use of 5–10 mm instruments.	Right and left colectomy and rectal resections, particularly in smaller operating rooms where arm modularity is advantageous.	Developed with lower acquisition and servicing costs in mind; expanding across UK and European public systems, including NHS hospitals.
**Hugo RAS (Medtronic)**	Open console with 3D display and separate hand controllers; facilitates team communication and allows flexible ergonomic setup.	Modular bedside arms on individual carts, mirroring laparoscopic port placement in a multi-port configuration.	Early series in colectomy and rectal resections; intended as a multispecialty platform for general, colorectal and urologic surgery.	Aims to compete on capital price and disposable costs; phased introduction in Europe, Latin America and parts of Asia.
**Hinotori (Medicaroid, Japan)**	Closed console with 3D vision and adjustable display/armrest.	Multi-arm cart system with wristed instruments; configuration optimised for pelvic and urologic procedures now expanding into colorectal indications.	Increasingly used for rectal cancer surgery in Japan within high-volume centres.	Represents a domestically developed alternative in a market with national insurance coverage for selected robotic procedures.
**Sentire Surgical System C1000 (Cornerstone Robotics)**	Closed immersive console with familiar layout and kinematics allowing for seamless transition from other closed- console systems for experienced robotic surgeons.	Multi-arm cart with four robotic arms, 3D HD vision and wristed instruments, mirroring established multi-port platforms.	Being evaluated for major gastrointestinal and colorectal procedures; early prospective data in radical prostatectomy show feasibility and comparable peri-operative outcomes.	Locally developed in Hong Kong with explicit focus on lowering capital and per-case costs in public systems; potential to improve access to robotics internationally.
**EndoMaster EASE (EndoMaster)**	Endoscopic-style console with dual robotic arms controlled to enhance traction and stability during endoscopic procedures, tailored to endoscopist ergonomics.	Flexible endoscope with two independently controlled articulated arms emerging near the tip, enabling bimanual submucosal dissection.	Colonic ESD for early colorectal neoplasia, expanding organ-preserving endoluminal treatment and potentially reducing need for formal resection in selected cases.	Currently available mainly within prospective trials at high-volume centres; if validated, may shift some early colorectal cancers from theatre to advanced endoscopy units.

This table compares major and emerging robotic platforms used in colorectal surgery globally, outlining console ergonomics, arm/port configuration, principal colorectal applications and key cost or access considerations.

## Data Availability

No new data were created.
